# Childhood cardiovascular disease risk profiles based on movement phenotypes:a longitudinal cohort study

**DOI:** 10.1007/s00431-025-06269-4

**Published:** 2025-06-19

**Authors:** Sami Yli-Piipari, Junhyuk Park, Sanga Yun, Yangyang Deng, Donna Niemistö, Iiris Kolunsarka, Mikko Huhtiniemi, Arto Gråstén, Timo Jaakkola

**Affiliations:** 1 Department of Kinesiology, 217 Ramsey Center, University of Georgia, 330 River Road, Athens, GA 30602 USA; 2 Department of Kinesiology, University of Georgia, Athens, 219 Ramsey Center, 330 River Road, GA 30602 USA; 3 Faculty of Sport and Health Sciences, University of Jyväskylä, PO Box 35, Jyväskylä, FI-40014 Finland; 4 Physical Education Department, United Arab Emirates University, PO Box 15551, Al Ain, UAE

**Keywords:** Adolescents, Latent profile, Obesity, Motor competence, Physical fitness, Physical activity

## Abstract

Cardiovascular disease (CVD) remains a significant global health concern, with many risk factors emerging in adolescence. This period is critical for prevention, as physical and behavioral patterns established during these years often persist into adulthood. Movement phenotypes, encompassing motor competence, physical capacity, and physical activity behaviours, are linked to cardiometabolic health as low competence and fitness levels in youth are associated with poor body composition and increased CVD risk. This longitudinal study aimed to (1) identify latent clusters of adolescents’ movement phenotype-related CVD risk factors and (2) examine the stability of these profiles over four years. Latent profile and transition analysis were used to identify movement phenotype profiles and transitions of cluster membership across time among 1,147 adolescents (*M*_age_: 11.27 ± .32). A four-cluster solution was identified as the most suitable. Profile 1 (23%) had the lowest motor competence, cardiovascular and muscular fitness, and moderate-to-vigorous physical activity (MVPA), along with the highest standardized body mass index (BMIz). Profile 2 (20%), predominantly girls, had below-average motor competence, cardiovascular and muscular fitness. The largest group, Profile 3 (36%), showed healthy indicators, with above-average values across all variables. Profile 4 (20%) had the highest levels of motor competence, cardiovascular and muscular fitness, and MVPA, as well as healthy BMIz (-2 ≤ BMIz ≤ 1). Cluster memberships remained remarkably stable over four years, except for a notable transition of over 20% from Profile 4 to 3. *Conclusion*: This study identifies distinct adolescent movement patterns associated with CVD risk and demonstrates how these change over time. The findings support the development of targeted interventions and early preventive strategies to support long-term cardiovascular health in adulthood.

**Table:** 

**What is Known – What is New** • Childhood movement phenotypes, i.e., motor competence, physical capacity, and behaviors, were highly stable over four years of adolescence, with nearly 50% of participants displaying elevated cardiovascular disease risk factors. • Additionally, 25% of our sample belonged to a cluster characterized by the poorest cardiovascular disease risk profile, marked by low motor competence, poor cardiovascular and muscle fitness, and low levels of moderate-to-vigorous physical activity. Most participants in this cluster also exhibited unhealthy body composition.

**Supplementary Information:**

The online version contains supplementary material available at 10.1007/s00431-025-06269-4.

## Introduction

Cardiovascular disease (CVD) poses a significant global health threat and has been the leading cause of death in the United States and worldwide for the past 100 years [1]. Overweight, obesity, and a lack of regular physical activity are among the key risk factors contributing to CVD [2]. Adolescence is a critical period for preventing CVD, as lifestyle-related behavioural habits established during this developmental stage often persist into adulthood and have a significant impact on long-term cardiovascular health [3]. Specifically, adolescent movement phenotypes play a crucial role in CVD prevention, as movement traits are closely linked to cardiovascular health [4, 5]. Despite growing interest in childhood risk factors for adult CVD, examination of latent movement phenotypes and their underlying patterns during childhood and adolescence remains limited, hindering the development of effective intervention strategies.

The Oxford Dictionary defines a phenotype as “the set of observable characteristics of an individual” [6]. In this study, movement phenotypes refer to observable movement-related traits, including motor competence (e.g., agility, coordination), physical capacity (e.g., cardiovascular and muscular fitness, and body composition), and behaviours (i.e., physical activity). These interrelated factors can collectively influence CVD risk directly and indirectly. For instance, childhood motor competence, i.e., the ability to perform motor skills effectively, is linked to increased physical activity participation [7, 8], higher intensity exercise [9], and healthier body mass index (BMI) [7, 10]. Motor competence is also positively associated with cardiovascular and muscular fitness, with this relationship strengthening over time [10–12]. Muscular fitness, in particular, plays a key role in cardiovascular health [13–15], with cardiovascular fitness shown to be a stronger predictor of cardiovascular health than physical activity behaviours [14]. These physical capacities are also associated with better glucose metabolic regulation [13, 15, 16] and a lower risk of overweight and obesity [17, 18]. Additionally, poor body composition is strongly related to clustered CVD risk in adolescence [19]. Finally, physical inactivity, one of the central CVD risk factors, negatively affects muscular fitness [20–22], body composition [23], and contributes to major CVD-related conditions such as myocardial infarction, stroke, heart failure, and type 2 diabetes mellitus [24].

Considering that movement competence, physical capacity, body composition, and physical activity behaviours are closely intertwined, understanding the roles of these movement phenotypes is crucial. However, research examining the formation and development of these movement phenotype-related CVD risk factors during adolescence has been limited. To address this gap, the aim of this study was twofold: 1) to identify and examine CVD risk profiles in children based on movement phenotypes, and 2) to examine the transition probabilities of these identified CVD risk profiles over time.

## Methods

### Participants

The study included 1,147 Finnish schoolchildren (51% of girls), representing two percent of the total population of 61,062 fifth graders at baseline [25]. Children were recruited from 35 randomly selected public schools in Southern (46% of students), Central (41%), Eastern (6%), and Northern Finland (7%). Written parental consent was sought and obtained to confirm their participation. The opportunity to participate was equally offered to all students, yet no children with disabilities or special needs participated. The study received approval from the University of Jyväskylä’s institutional review board and adhered to the ethical guidelines for human participant research set forth in the Declaration of Helsinki.

### Procedure

Data were collected using identical procedures between August and September in 2017 (Time 1; T1; *n* = 1,147), 2019 (Time 2; T2; *n* = 885), and 2021 (Time 3; T3; *n* = 738). Participants self-reported their demographic and physical activity information in a classroom setting. Motor competence, fitness, and anthropometric data were collected by trained physical education teachers in the school gym using a standardized protocol.

### Measures

#### Motor competence

Participants’ motor competence was assessed using the following tests: (a) side-to-side jumping test, (b) throwing-catching combination test, and (c) 5-leaps test, all of which have acceptable validity and reliability for children and adolescents [26]. In the side-to-side jumping test, participants were asked to jump side-to-side over a beam (60 × 4x2 cm) for as many times as they could in 15 s, with their feet together. The final score was the average score of two test attempts. For the throwing-catching combination test, participants were instructed to throw a tennis ball directly at a designated target area (1.5 × 1.5 m, 90 cm above the floor) and catch the ball as it bounced back after hitting the target and the floor. The score was the total number of successful attempts out of 20 trials. Lastly, in the 5-leaps test, participants were asked to leap five times as far as possible, starting their first jump and landing their fifth jump with their feet parallel. The 5-leap sequence consisted of alternating leaps, starting with their preferred leg, followed by a jump with the opposite leg. The score was the total leap distance measured in centimetres. The composite was created by converting each raw score into z-scores and subsequently averaging the scores into one composite score.

#### Cardiovascular endurance

The progressive aerobic cardiovascular endurance run (PACER) test [27], which has been shown to be valid and reliable [28], was used to assess participants’ cardiovascular endurance. Following the guidance of a recorded cadence, participants were instructed to run as many laps as possible until they could no longer keep pace with the cadence. Each lap required running between two parallel lines 20 m apart. The final score was the number of completed laps.

#### Muscle strength/endurance

Participants’ muscle strength and endurance were measured using their (a) curl-up and (b) push-up test scores, which have been validated and found reliable for children and adolescents [26]. For the curl-up test, participants were asked to lie on their backs with their knees bent at 100° and their feet flat on the floor. A measuring tape was placed under the participants so that their fingertips touched the nearest edge of the tape with their arms straight and palms straight on the floor. They were instructed to curl up until their fingertips slid to touch the other end of the tape, following a cadence. The final score was the total number of correctly completed curl-ups, with a maximum score of 75 repetitions. In the push-up test, boys performed push-ups with their hands and feet on the floor while girls performed a modified version with their knees on the floor. With their body and legs straight in line, arms shoulder-width apart, and their feet (boys) or knees (girls) together, they were asked to lower their body until their upper arms were parallel to the floor and then push back up. The score was the number of correctly completed push-ups in 1 min. The composite score was calculated by standardizing each raw test score and computing the average.

#### Body composition

BMI (kg/m.^2^) was calculated. Height was measured to the nearest 0.1 cm using a stadiometer, and weight was measured to the nearest 0.1 kg using a digital scale, with participants wearing light clothing and no shoes. Standardized BMI (BMIz) was calculated using an SPSS macro, which has been shown to be valid and reliable [29, 30].

#### Physical activity

Participants’ health-enhancing physical activity was assessed by self-reported moderate to vigorous physical activity (MVPA) using the International Physical Activity Questionnaire. This questionnaire asks participants to recall the number of days and minutes per day they engaged in MVPA for the past seven days. Weekly MVPA (min/week) was calculated by summing moderate and vigorous activity minutes. The scale has demonstrated moderate reliability and validity for estimating total PA in adolescents [31].

#### Covariates

Age was determined by subtracting the date of birth from the measurement date and then converting the result into years. Biological sex was classified as male or female based on birth sex. Peak height velocity (PHV) was included as a covariate to account for variations in biological maturation during puberty, which can influence adolescents’ physical capacity, motor competence, and body composition. The maturity offset was calculated using an equation that considers the documented age and height at each measurement [32]. This offset reflects how close a child is to reaching PHV by subtracting the child’s chronological age from the age at which PHV occurs. A negative offset means the child has not yet reached PHV. If the offset is positive and greater than 1.5, PHV has already occurred; if it is positive but less than 1.5, the child is still in the process of reaching PHV.

### Data analysis

Latent profile analysis is a probabilistic modelling algorithm that allows clustering of data and statistical inference to split potentially heterogeneous data into subclasses of homogeneous clusters [33–35]. This operates on the assumption that the observed variable distributions are the result of a finite latent mixture of underlying distributions [33]. Latent profiles were identified to enhance our understanding of the patterns of risk factors contributing to an elevated risk of CVD [36]. Based on these patterns, latent transition analysis was conducted to estimate the probabilities of transitions among profiles over time.

Prior to conducting the primary analyses, the data were checked for normality, outliers, and missing values. Descriptive statistics were reviewed for each time point. Latent profile analysis was performed to identify latent clusters based on outcome variables. Latent transition analysis, following the five-step protocol [37], was used to examine transition probabilities between clusters, accounting for covariate effects of sex, age, and PHV. The steps in the analysis were: 1) Diagnosing cross-sectional data and identifying clusters at each time point using latent profile modelling; 2) Testing longitudinal measurement invariance between clusters identified via latent transition modelling; 3) Defining latent clusters and calculating cluster-specific statistics; 4) Assessing transition probabilities and invariance between clusters; and 5) Evaluating covariate effects at each time point. The most appropriate latent cluster solution was determined using several criteria: the Akaike Information Criterion (AIC), Bayesian Information Criterion (BIC), sample-size adjusted BIC (ABIC), Adjusted Lo-Mendell-Rubin likelihood ratio test (ALMR-LRT), and entropy values [38]. A lower AIC, BIC, and ABIC, along with higher entropy, indicated a better model fit. The ALMR-LRT test assessed the fit of the current model by comparing it to a model with one fewer cluster, favouring the current model if it showed a statistically significant improvement. A *p*-value of less than 0.05 was considered statistically significant for all analyses. Measurement invariance, where intercepts are fixed across time in all clusters, was tested using the unconstrained model to ensure that the latent clusters were distinctly defined. After identifying the best cluster solutions over time, transition probabilities and their invariance between latent clusters were analysed. Finally, the effects of covariates on latent cluster membership at each time point were evaluated. Data checks for normality, outliers, and missing values were conducted using SPSS 29.0, while subsequent analysis was performed with Mplus 8.10.

## Results

### Preliminary analyses

Graphical examination using Quantile–Quantile plots indicated that the observed variables were approximately normally distributed, with no extreme skewness or kurtosis, and standardized values within ± 3.0, suggesting no outliers. The percentage of missing values was 25% (8,720 out of 34,410 values). The missing values were due to a decrease in the proportion of students participating in follow-up measures over time (Table 1). The Missing Completely at Random (MCAR) test [39] indicated (χ^2^ = 9,972.72, *df* = 9,765, *p* = 0.069) that the estimated full data matrix and the current incomplete data matrix with missing values were equal. Therefore, no further data modification due to the missing scores was executed. As Little’s MCAR test suggested that the pattern of missingness did not deviate from MCAR, we used full-information maximum likelihood (FIML) in all subsequent analyses. FIML incorporates all available data points from each participant when estimating model parameters, producing unbiased estimates and standard errors under the MCAR assumption, while preserving sample size and maximizing statistical power [40]. Descriptive statistics were computed in Table 1. The sample was 11-year-old (M = 11.27 ± 0.32) pre-pubertal children, an average of 1.30 years from the PHV (M = −1.30 ± 0.77) at the start of the study.

**Table 1:** Participants’ minimum and maximum scores, means, and standard deviations of the study variables at each time point

		*N*	Min	Max	*M*	*SD*
Motor competence	T1	1,117	−3.15	2.03	−0.01	0.80
	T2	885	−2.49	2.92	0.01	0.83
	T3	567	−3.08	3.19	−0.01	0.83
Side-to-side	T1	1,089	11	56	37.27	6.55
	T2	848	16	62	44.40	7.13
	T3	527	12	67	47.60	8.14
Throwing-catching	T1	1,106	0	20	10.40	5.28
	T2	862	0	20	10.95	4.89
	T3	559	0	20	13.40	4.63
5-leap	T1	1,099	3.70	10.06	7.74	0.89
	T2	838	5.52	11.60	8.58	1.09
	T3	539	3.40	13.93	9.31	1.41
Muscle strength/endurance	T1	1,106	−1.77	3.04	−0.01	0.84
	T2	866	−1.91	2.38	0.01	0.86
	T3	548	−2.19	3.19	−0.01	0.88
Curl-up	T1	1,074	0	75	37.85	21.87
	T2	841	0	75	39.98	21.32
	T3	526	1	75	46.67	22.66
Push-up	T1	1,070	0	75	21.58	12.22
	T2	844	0	75	25.56	13.19
	T3	501	0	72	29.38	13.38
Cardiovascular endurance	T1	1,057	1	94	36.06	18.33
	T2	765	1	103	39.10	19.58
	T3	436	1	107	40.91	22.14
Self-reported MVPA	T1	1,086	1	7	4.99	1.58
	T2	875	1	7	5.14	1.55
	T3	738	0	7	4.73	1.74
BMI (kg/m^2^)	T1	1,120	13.54	36.35	18.89	3.12
	T2	836	14.53	35.95	20.32	3.36
	T3	578	14.57	36.52	21.44	3.21
BMIz	T1	1,106	−2.47	3.89	0.46	1.09
	T2	817	−2.39	3.45	0.39	1.05
	T3	563	−2.89	3.28	0.28	0.99
PHV	T1	1,106	−2.93	0.61	−1.30	0.77
	T2	839	−1.39	2.91	0.54	0.90
	T3	577	0.33	5.41	2.24	0.80
Age	T1	1,147	10.69	12.64	11.27	0.32
	T2	885	12.70	14.70	13.28	0.33
	T3	738	14.70	16.70	15.28	0.33

Time 1, *T1* Time 2, Time 3, *T3* Peak height velocity: *PHV*

### Latent profile analysis

Latent cluster memberships were estimated at each time point and across all time points, as presented in Table 2. The 4-cluster structure was favoured at all time points. At Time 1, both 3-cluster and 4-cluster solutions appeared reasonable, because the 4-cluster had the highest entropy and lower AIC and BIC values than the 3-cluster, while profile 4 had fewer than 5% of the sample. However, after evaluating model fit indices across all time points, the 4–4-4 cluster solution was considered the most appropriate, demonstrating a better fit than the 3–4-4 solution and yielding a satisfactory entropy value of 0.8. Based on this totality of the fit, the 4-cluster solution was determined as the most justifiable over time.

**Table 2:** The parameter estimates for the latent profile solutions within one to five groups

	Parameters	AIC	BIC	ABIC	LT5%	LT1%	pLMR	Entropy
T1								
1-solution	10	22,002	22,053	22,021	-	-	-	-
2-solution	16	21,051	21,132	21,081	-	-	0.000	0.72
**3-solution**	**22**	**20,809**	**20,920**	**20,850**	**-**	**-**	**0.004**	**0.71**
**4-solution**	**28**	**20,730**	**20,871**	**20,782**	**1**	**-**	**0.007**	**0.74**
5-solution	34	20,664	20,835	20,727	-	-	0.098	0.69
T2								
1-solution	10	16,738	16,787	16,755	-	-	-	-
2-solution	16	15,791	15,869	15,819	-	-	0.000	0.69
3-solution	22	15,357	15,465	15,395	-	-	0.000	0.73
**4-solution**	**28**	**15,172**	**15,309**	**15,220**	**-**	**-**	**0.037**	**0.74**
5-solution	34	15,070	15,237	15,129	1	-	0.042	0.73
T3								
1-solution	10	11,277	11,325	11,293	-	-	-	-
2-solution	16	10,734	10,810	10,759	-	-	0.000	0.56
3-solution	22	10,536	10,640	10,570	-	-	0.006	0.58
**4-solution**	**28**	**10,445**	**10,577**	**10,488**	**-**	**-**	**0.027**	**0.60**
5-solution	34	10,416	10,577	10,469	1	-	0.281	0.60
Model	G^2^	AIC	BIC	Entropy	df	Diff. G^2^	Diff. df	p
3–3-3 (Unconstrained) Non	−22,831	45,809	6182	0.74	74	83	30	0.000
3–3-3 (Constrained)	−22,914	45,916	46,138	0.73	44			
**4–4-4 (Unconstrained)**	**−22,573**	**45,350**	**45,865**	**0.80**	**102**	**93**	**40**	**0.000**
4–4-4 (Constrained)	−22,666	45,457	45,770	0.77	62			
3–4-4 (Unconstrained)	−22,677	45,541	46,010	0.70	93	79	35	0.000
3–4-4 (Constrained)	−22,756	45,628	45,921	0.72	58			

Bold indicates the most reasonable solution at each time point. *AIC*Akaike Information Criterion, *BIC* Bayesian Information Criterion, *ABIC* Adjusted Bayesian Information Criterion, *LT* less than, *pLMR* *p*-value for Adjusted Lo-Mendell-Rubin Ratio Test, *G*^2^ likelihood ratio, *df* degrees of freedom; *Diff. G*^2^ likelihood ratio difference, *Diff. df* degrees of freedom difference

**Fig. 1:**
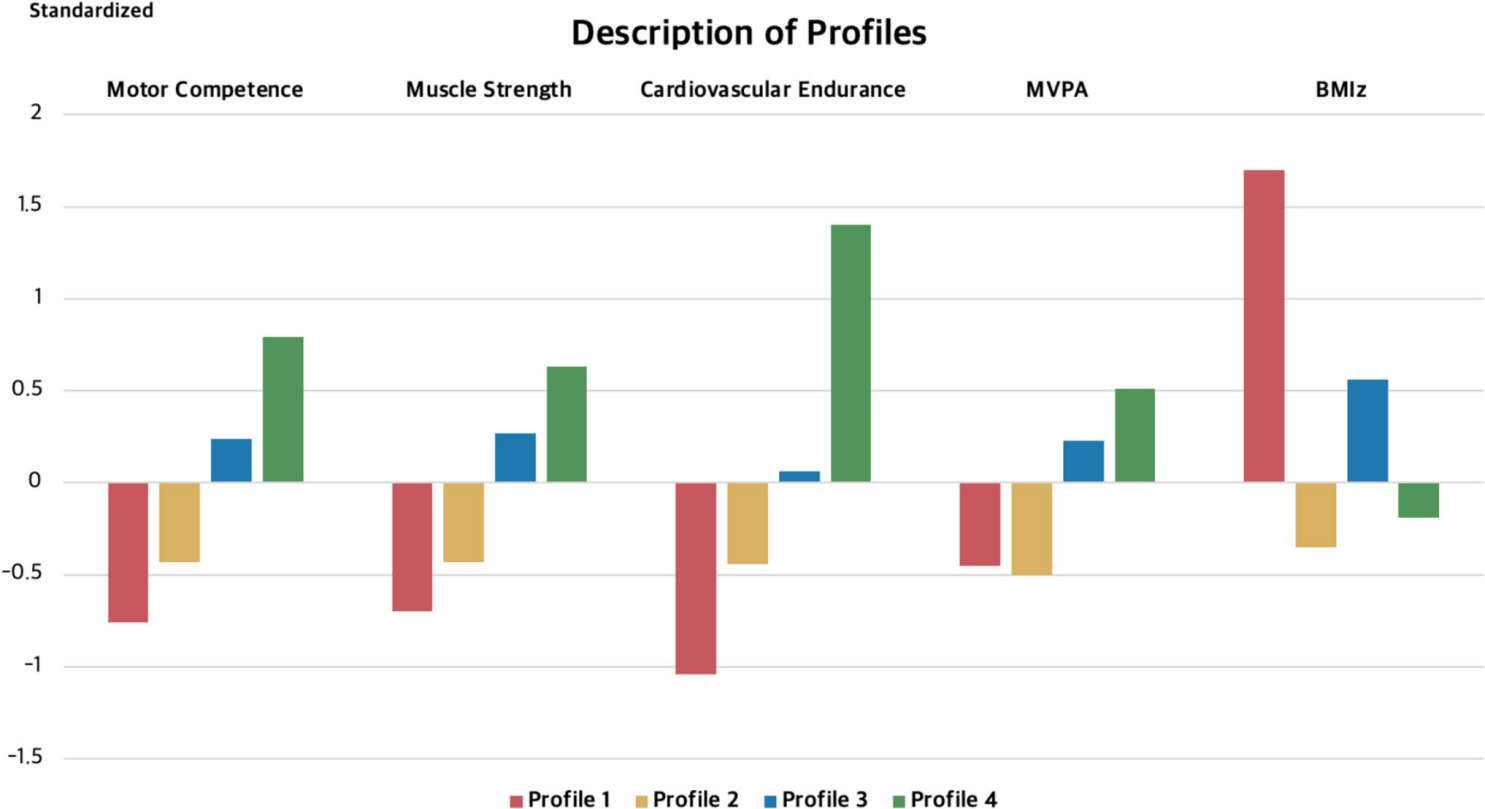
Characteristics of movement phenotype profiles

Profile 1 comprised 23% of the sample and had the lowest motor competence, cardiovascular and muscular fitness, MVPA, and the highest BMIz compared to other clusters. This group showed a less favourable CVD risk profile, characterized by low motor competence and high BMIz. Profile 2 included nearly 20% of the sample. These participants had generally below-average motor competence, cardiovascular fitness, and muscular fitness, though their levels were better than Profile 1. This group was the least physically active, but they had lower BMIz scores. A high proportion of participants in this profile were girls (T1 62%, T2 62%, and T3 61%). The largest number of students belonged to Profile 3 (around 36%). They exhibited healthy indicators, with above-average motor competence, cardiovascular and muscular fitness, and MVPA. Finally, Profile 4 comprised around 21% of the sample, including participants with the highest levels of motor competence, cardiovascular and muscle fitness, MVPA, as well as healthy BMIz (−2 ≤ BMIz ≤ 1). Most participants in Profile 4 were boys (T1 66%, T2 67%, and T3 66%). Figure 1 presents standardized scores for key variables across four clusters. There were no differences in participants’ age or physical maturity at any time point.

Longitudinal measurement invariance over time was tested to avoid ambiguity when defining latent statuses (Table 2). As the AIC, BIC, and entropy indices indicated that the 4–4-4 unconstrained model provided the best fit, unconstrained (freely estimated) and constrained (intercepts fixed to be equal across time) versions of the 4–4-4 model were compared. The freely estimated and constrained models were unequal, but the constrained model’s fit indices were not worse than those of the unconstrained model, indicating evident measurement invariance across clusters. Despite unequal intercepts, comparable model fit supports consistent latent status definitions across time points, justifying the 4–4-4 cluster solution as the most reasonable. Means and standard deviations of the study variables by clusters and the status prevalence within girls and boys are presented in Table 3.

**Table 3:** Means and standard deviations of the study variables by clusters and the status prevalence within girls and boys

		Profile1 *M* (*SD*)			Profile 2 *M* (*SD*)			Profile 3 *M* (*SD*)			Profile 4 *M* (*SD*)			
Motor competence*	T1	−0.76 (0.65)			−0.43 (0.58)			0.24 (0.52)			0.79 (0.52)			
	T2	−0.90 (0.55)			−0.38 (0.48)			0.16 (0.57)			0.92 (0.57)			
	T3	−0.68 (0.59)			−0.32 (0.50)			0.08 (0.74)			0.62 (0.85)			
Side-to-side	T1	31.93 (5.69)			34.56 (5.22)			39.16 (5.32)			42.17 (5.23)			
	T2	37.21 (6.11)			41.90 (5.95)			46.56 (5.27)			50.59 (50.01)			
	T3	41.80 (7.57)			46.47 (6.13)			48.65 (7.36)			51.87 (8.34)			
Throw-catch	T1	7.58 (4.83)			7.49 (4.80)			11.48 (4.51)			14.36 (3.99)			
	T2	8.39 (5.08)			8.90 (4.61)			11.94 (4.14)			14.19 (3.72)			
	T3	10.57 (4.94)			11.64 (4.42)			14.18 (3.99)			16.26 (3.26)			
5-jump	T1	6.91 (0.70)			7.46 (0.70)			7.95 (0.67)			8.51 (0.72)			
	T2	7.57 (0.87)			8.24 (0.96)			8.85 (0.85)			9.51 (0.82)			
	T3	8.33 (1.32)			9.01 (1.14)			9.36 (1.26)			10.31 (1.28)			
Muscle strength*	T1	−0.70 (0.63)			−0.43 (0.56)			0.27 (0.72)			0.63 (0.75)			
	T2	−0.75 (0.67)			−0.44 (0.50)			0.22 (0.71)			0.75 (0.71)			
	T3	−0.60 (0.78)			−0.54 (0.62)			0.17 (0.74)			0.67 (0.76)			
Curl-up	T1	26.21 (18.61)			27.18 (16.06)			43.39 (20.89)			50.24 (21.21)			
	T2	24.28 (14.47)			29.41 (14.52)			46.48 (19.95)			56.46 (19.10)			
	T3	33.10 (18.55)			34.99 (18.90)			51.87 (20.85)			61.77 (19.69)			
Push-up	T1	11.33 (8.91)			17.22 (9.25)			25.15 (10.72)			30.11 (10.95)			
	T2	11.67 (9.27)			20.34 (9.13)			30.46 (9.15)			37.07 (11.31)			
	T3	18.94 (9.89)			21.73 (9.60)			32.76 (11.08)			40.22 (11.85)			
Cardio*	T1	17.06 (7.55)			27.96 (10.89)			37.18 (10.32)			61.74 (11.18)			
	T2	19.91 (9.17)			31.57 (11.35)			40.20 (12.37)			67.83 (12.76)			
	T3	24.59 (14.94)			34.23 (15.62)			42.39 (18.38)			60.85 (23.47)			
MVPA*	T1	4.26 (1.62)			4.20 (1.46)			5.36 (1.37)			5.79 (1.35)			
	T2	4.45 (1.64)			4.25 (1.52)			5.50 (1.29)			6.07 (1.06)			
	T3	4.25 (1.75)			3.86 (1.63)			5.03 (1.57)			5.66 (1.54)			
BMI	T1	22.53 (3.25)			16.79 (1.54)			18.87 (2.20)			17.03 (1.64)			
	T2	24.16 (3.51)			18.19 (1.96)			20.32 (2.44)			18.42 (1.90)			
	T3	24.52 (3.66)			19.37 (2.04)			21.62 (2.73)			20.19 (1.96)			
BMIz*	T1	1.70 (0.78)			−0.35 (0.76)			0.56 (0.80)			−0.19 (0.81)			
	T2	1.54 (0.80)			−0.41 (0.81)			0.57 (0.79)			−0.17 (0.76)			
	T3	1.20 (0.85)			−0.48 (0.78)			0.44 (0.85)			−0.03 (0.81)			
PHV	T1	−1.28 (0.76)			−1.23 (0.74)			−1.19 (0.78)			−1.57 (0.75)			
	T2	0.47 (0.86)			0.74 (0.83)			0.66 (0.87)			0.20 (0.94)			
	T3	2.20 (0.77)			2.38 (0.80)			2.36 (0.75)			1.93 (0.85)			
Age	T1	11.24 (0.33)			11.26 (0.31)			11.27 (0.33)			11.28 (0.32)			
	T2	13.25 (0.33)			13.27 (0.31)			13.28 (0.33)			13.29 (0.32)			
	T3	15.25 (0.33)			15.27 (0.31)			15.29 (0.33)			15.29 (0.32)			
Status prevalence**		Girls	Boys	All	Girls	Boys	All	Girls	Boys	All	Girls	Boys	All	
T1		121 21%	137 24%	258 23%	143 25%	89 16%	232 20%	237 40%	181 32%	418 36%	81 14%	158 28%	239 21%	
T2		124 21%	154 27%	278 24%	147 25%	91 16%	238 21%	241 42%	178 32%	419 36%	70 12%	142 25%	212 19%	
T3		134 23%	143 26%	277 24%	152 26%	98 17%	250 22%	234 40%	205 36%	439 38%	62 11%	119 21%	181 16%	

*Variables are included in the latent profile analysis

** Although the sample sizes at time points 2 and 3 are 885 and 738 respectively, FIML leveraged all available data from the full sample (*N* = 1,147) to estimate model parameters. This method used each participant’s available data, even if missing at some time points, to inform latent transitions, assuming that data are missing at random

### Latent transition analysis

**Fig. 2:**
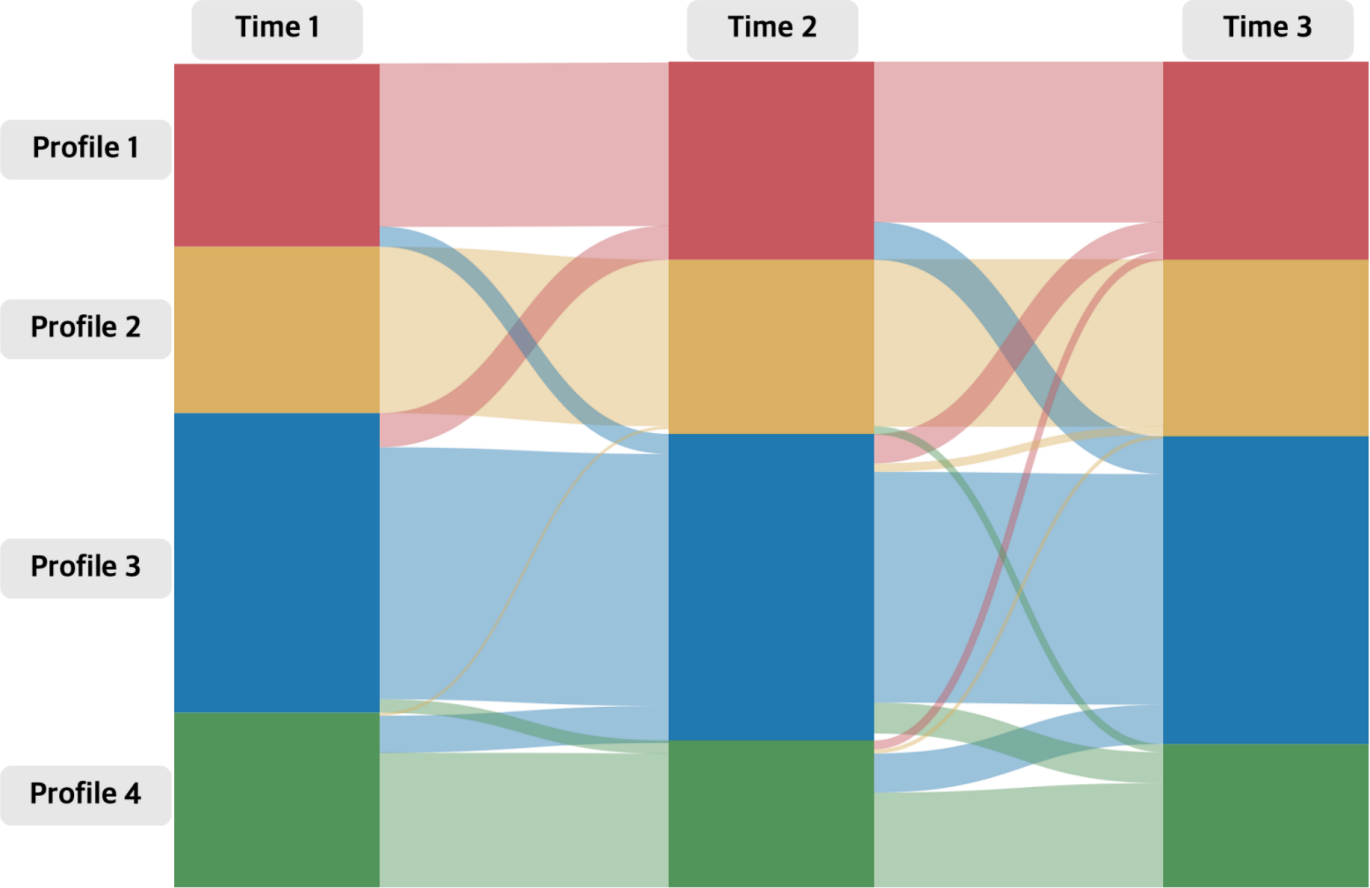
Transition probabilities between profiles across the three timepoints

The measurement invariance of transition probabilities over time was tested (Table 4). The unconstrained transition probability model (model 0) and the constrained model (model 1) were unidentical when the probabilities were fixed to be equal over time, indicating a significant variation in transition probabilities between clusters. However, the transition probabilities were relatively low over time, indicating that the cluster memberships remained stable from T1 to T3. For instance, Profile 2 members stayed 100% in the same cluster from T1 to T2. In addition, Profile 1 members (89.5%), Profile 3 members (84.1%), and Profile 4 members (77.2%) also showed high cluster stability from T1 to T2. Notably, there was significant movement from Profile 4 to Profile 3 at both time points: 21.1% from T1 to T2 and 24.8% from T2 to T3. Transition trends are illustrated in Fig. 2.

**Table 4:** Transition matrix estimates of class analysis-based clusters over three time points and transition probability invariance

τ T1-T2						τ T2-T3				
	Profile 1	Profile 2		Profile 3 33 3	Profile 4		Profile 1	Profile 2	Profile 3 3 33 3	Profile 4
Profile 1	**0.895**	0.000		0.105	0.000	Profile 1	**0.811**	0.000	0.189	0.000
Profile 2	0.000	**1.000**		0.000	0.000	Profile 2	0.000	**0.953**	0.000	0.047
Profile 3	0.114	0.000		**0.841**	0.045	Profile 3	0.099	0.028	**0.770**	0.103
Profile 4	0.000	0.017		**0.212**	**0.772**	Profile 4	0.056	0.026	**0.248**	**0.670**
Invariance i		G^2^	AIC		BIC	Entropy	df	Diff. G^2^	Diff. df	*p*
Model 0 (unconstrained)		−22,573	45,350		45,865	0.80	102	71	8	0.000
Model 1 (constrained)		−22,644	45,477		45,952	0.71	94			

*τ* Transition estimates, *G2* likelihood ratio, *AIC* Akaike Information Criterion, *BIC* Bayesian Information Criterion, *df* degrees of freedom, *Diff. G2* likelihood ratio difference, *Diff. df* degrees of freedom difference. Bold indicates the probability > 0.20

In the final step, the covariates of sex, age, and PHV were added to the 4–4-4-transition model to examine their effects on the cluster membership prevalence at each time point. In the case of the multinomial model, the statistical program defined an estimated value of 0 for the reference group (Profile 4). Girls were more likely to be members of Profile 2 at T1 (β = −2.91[1.18]) and Profile 1 at T3 than in Profile 4 (β = −2.62[1.41]). Although all the students in the sample were in the same grade at each time point, those born earlier in the year were more likely to be in Profile 3 than Profile 4 at T1 (β = 1.85[0.84]). Finally, the students with lower maturity offset had a higher probability of being in Profiles 3 (β = −2.05[0.78]) and 2 (β = −1.86[0.77]) at T1 than in Profile 4.

## Discussion

This study contributes to the current literature on CVD risk factors by examining how movement phenotype-related risk profiles develop and persist across adolescence. Previous studies using the latent profile or class approach have also identified 3- to 4-cluster solutions to classify CVD risk groups, though based on different combinations of risk factors than those used in our study. For example, Tegegne et al. [41] identified three lifestyle risk clusters among at-risk adults and four clusters among adults with CVD, based on smoking, physical inactivity, unhealthy diet, and alcohol consumption. Furthermore, CVD risk factor profiles have been clustered into three groups among elderly and patient populations based on physical activity types (occupational, sedentary, and leisure-time) by Chen et al. [42], and on demographic factors (race, age, and sex) by Kundi et al. [43]. Building on this prior work, our study extends the application of clustering approaches by suggesting movement phenotypes as CVD-related risk profiles specifically among adolescents for the first time.

It is particularly concerning that nearly 50% of participants fell into Profiles 1 and 2, as this high proportion represents a significant public health issue. These individuals are at greater risk of developing cardiovascular problems over time due to low motor competence, poor fitness, and insufficient physical activity. The elevated BMI in Profile 1 further exacerbates this risk. These findings align with previous correlational research evidence showing that children and adolescents with obesity often have low motor competence [44]. In addition, the study by Chagas et al. [45] has shown that adolescents with low motor competence are six times more likely to become overweight or obese in adulthood.

Despite some variations in transition probabilities between clusters, membership remained surprisingly consistent throughout adolescence. While most participants stayed in the same cluster over time, about one-fifth to one-fourth of those in Profile 4 shifted to Profile 3. These findings suggest, firstly, that unhealthy behaviours are stable during critical adolescent years. Although students born earlier in the year were more likely to be included in Profile 4, maturity did not influence profile transitions over time. While the data were MCAR and handled using FIML, missing data may still have impacted the accuracy of stability estimates due to unmeasured factors related to attrition.

Second, a large portion of highly active adolescents with healthy movement behaviours experience a decline in their motor competence and physical capacity. This suggests that even individuals who regularly engage in MVPA and appear healthy may be at risk of deteriorating motor skills and fitness levels. To the authors’knowledge, few studies have examined the stability of CVD risk profiles over time. Although based on a different age group and health context, Steca et al. [46] found that individuals in a poor lifestyle profile experienced the greatest difficulty changing their health behaviours after an acute coronary event. Notably, the stability of profiles observed as early as age 11 in our study suggests that unhealthy patterns may already be well established by that point. This underscores the importance of implementing preventive interventions within families and schools even before this age, as lifestyle behaviours developed in childhood have been shown to track into adulthood [47].

Our study findings have important clinical implications, as they can guide the development of targeted interventions and tailored strategies to address CVD risk factors from an early age, ultimately supporting improved cardiovascular health outcomes into adulthood. Specifically, interventions can target the two poorer profiles through early screening and behavior improvement while focusing on monitoring and maintaining healthy behaviours in the two better profiles. For instance, behavior improvement can be achieved by enhancing motor competence through motor skill learning opportunities, which in turn fosters greater confidence, motivation for physical activity, and overall behavioural improvements. Maintenance strategies may include ongoing encouragement of long-term engagement in sports and active play across school, family, and community settings. Particularly, as the observed stability of risk patterns indicates they are unlikely to change without intentional support, this reinforces the importance of early, individualized prevention strategies in public health, education, and clinical practice. Subsequent studies should explore the underlying mechanisms driving transitions between profiles, investigate the long-term health outcomes associated with different movement phenotypes, and evaluate the effectiveness of early interventions aimed at modifying risk trajectories.

This study has several limitations. First, it did not include direct clinical markers of cardiovascular health, such as blood pressure or lipid profiles, limiting our ability to directly assess physiological risk. Instead, the focus was on movement phenotype-related risk factors that are established precursors of CVD later in life. Future research should incorporate clinical health measures to strengthen the link between early movement patterns and long-term cardiovascular outcomes. Second, although missing data were confirmed to follow an MCAR pattern and were handled using FIML, attrition over time inevitably reduced the sample size and may have limited statistical power. Longitudinal designs that utilize strategies to minimize attrition can be crucial for future studies. Third, while movement phenotypes were central to our framework, other important contributors to cardiovascular risk, such as socioeconomic status, diet, and access to healthcare, were not included [48]. Incorporating these variables would offer a more comprehensive understanding of CVD risk development. Finally, the findings may not be generalizable to other populations, as the sample consisted exclusively of Finnish adolescents. Future studies with more diverse and international samples are needed to evaluate the generalizability of movement phenotype profiles across different demographic and cultural contexts.

## Supplementary Information

Below is the link to the electronic supplementary material.

Supplementary file 1 (DOCX 17.5 KB)

Supplementary file 2 (DOCX 262 KB)

## Data Availability

Data is provided within the manuscript.

## Abbreviations

CVD: Cardiovascular disease
BMI: Body mass index
BMIz: Standardized body mass index
MVPA: Moderate to vigorous physical activity
PHV: Peak height velocity
