# Supplementary material for: Childhood cardiovascular disease risk profiles based on movement phenotypes:a longitudinal cohort study
Source: Eur J Pediatr. 2025 Jun 19;184(7):428. doi: 10.1007/s00431-025-06269-4 (PMC12176961; doi:10.1007/s00431-025-06269-4)
Supplement: Supplementary file 1 — (DOCX 17.5 KB) [file 431_2025_6269_MOESM1_ESM.docx]

*Appendix 1. Attrition analysis*

| Variable | Mean (Retained) | Mean (Dropped) | *t*-value (*p*-value) |
| --- | --- | --- | --- |
| BMIz | 0.42 | 0.50 | 1.21 (0.23) |
| Side-to-side | 37.81 | 36.94 | -2.14 **(0.03)** |
| Throwing-catching | 10.82 | 10.14 | -2.09 **(0.01)** |
| 5-leaps test | 7.76 | 7.73 | -0.59 (0.56) |
| PACER | 37.27 | 35.31 | -1.72 (0.09) |
| Curl-up | 37.9 | 37.82 | -0.06 (0.95) |
| Push-up | 20.41 | 22.33 | 2.53 **(0.01)** |
| MVPA | 5.18 | 5.25 | 0.62 (0.53) |

*Note.* Attrition analysis suggests that, although most baseline variables did not differ significantly between groups, a few indicators (particularly those related to motor competence and muscular fitness) show statistically significant differences. While this may suggest some degree of attrition bias, the practical significance of these differences should be considered. In large samples, even small differences can reach statistical significance without being practically meaningful. For instance, the mean difference between the retained and dropped groups was less than one repetition in both the side-to-side jumping test and throwing-catching test, and approximately two repetitions in the push-up test.
