# Supplementary material for: Childhood cardiovascular disease risk profiles based on movement phenotypes:a longitudinal cohort study
Source: Eur J Pediatr. 2025 Jun 19;184(7):428. doi: 10.1007/s00431-025-06269-4 (PMC12176961; doi:10.1007/s00431-025-06269-4)
Supplement: Supplementary file 2 — (DOCX 262 KB) [file 431_2025_6269_MOESM2_ESM.docx]

**
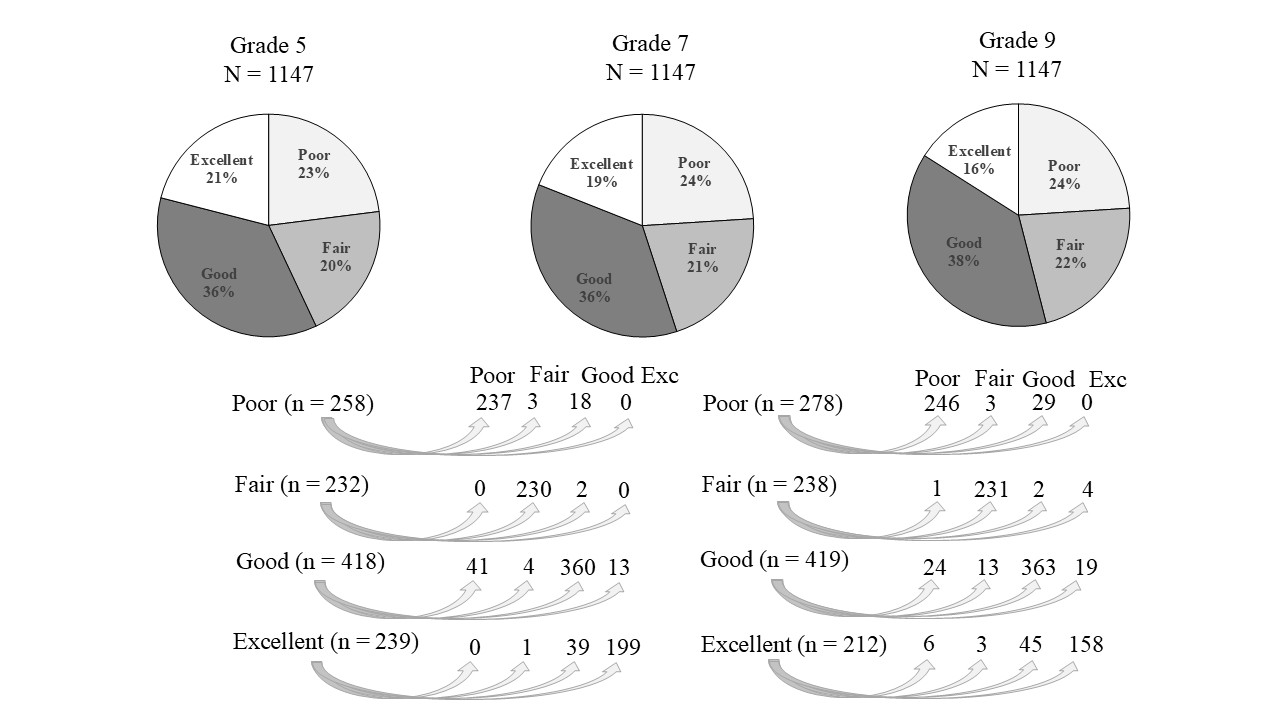
**

*Note.* Profiles are categorized as follows: Profile 1 = Poor, Profile 2 = Fair, Profile 3 = Good, Profile 4 = Excellent.

*Appendix 2. Profile distribution and transition across time points.*
